# Perceptions of Undue Influence Shed Light on the Folk Conception of Autonomy

**DOI:** 10.3389/fpsyg.2018.01400

**Published:** 2018-08-08

**Authors:** Fay Niker, Peter B. Reiner, Gidon Felsen

**Affiliations:** ^1^Department of Politics and International Studies, University of Warwick, Coventry, United Kingdom; ^2^National Core for Neuroethics, Department of Psychiatry, The University of British Columbia, Vancouver, BC, Canada; ^3^Department of Physiology and Biophysics, University of Colorado School of Medicine, Aurora, CO, United States; ^4^Center for Bioethics and Humanities, University of Colorado School of Medicine, Aurora, CO, United States

**Keywords:** agency, autonomy, behavioral control, decision making, experimental philosophy, nudging

## Abstract

Advances in psychology and neuroscience have elucidated the social aspects of human agency, leading to a broad shift in our thinking about fundamental concepts such as autonomy and responsibility. Here, we address a critical aspect of this inquiry by investigating how people consider the socio-relational nature of their own agency, particularly the influence of others on their perceived control over their decisions and actions. Specifically, in a series of studies using contrastive vignettes, we examine public attitudes about when external influences on everyday decisions are perceived as “undue” – that is, as undermining the control conditions for these decisions to be considered autonomous – vs. when they are perceived as appropriate and even supportive of autonomous decision-making. We found that the influence of preauthorized agents – individuals and institutions with whom we share a worldview – was judged to be less undue than non-preauthorized agents, even after controlling for the familiarity of the agent. These effects persisted irrespective of the extent to which respondents identified as communitarian or individualistic, and were consistent across two distinct scenarios. We also found that external influences that were rational were perceived as less undue than those that were arational. Our study opens new avenues of inquiry into the “folk conception” of autonomy, and we discuss the implications of our findings for the ethics of public policies designed to influence decisions and for information sharing in social networks.

## Introduction

Recent discoveries in psychology and neuroscience have set in motion a broad shift in our understanding of human agency. Traditionally, our concept of agency has hinged on rational thinking under the exclusive domain of the individual. Such conceptions are called into question by findings that highlight the automatic and social dimensions of our cognition. Under certain circumstances, our thinking and action tends to be “embedded in and dependent upon (features of our) social environment” (Hurley, 2011, p. 192). This raises important questions about the capacity to control one’s behavior in such cases and, relatedly, about how we should think about central human values such as freedom, autonomy, and responsibility.

There is a burgeoning philosophical literature on these topics. For instance, some theorists have argued in favor of reconceptualizing personal autonomy along socio-relational lines (Mackenzie and Stoljar, 2000; Oshana, 2006), and the implications of this reconceptualization for other concepts in moral and political theory are beginning to be considered (Christman, 2014; Specker Sullivan and Niker, 2018). There is also increasing philosophical interest in exploring and integrating social dimensions into our accounts of moral responsibility (Vargas, 2013; Hutchison et al., 2018).

In this paper, we focus on a distinct but complementary aspect of this inquiry: examining how people consider the socio-relational nature of their own agency, and particularly the influence of others on their perceived control over their decisions and actions. Specifically, our study investigates public attitudes about when certain kinds of external influences on decisions are perceived as “undue” – that is, as something that undermines the control conditions for these decisions to be considered autonomous – vs. when they are perceived as appropriate and even supportive of decision-making.

While there is general consensus on the “undueness” of heavy-handed forms of influence like brainwashing and manipulation (Birks and Chin, 1995), we are interested in how people perceive of more everyday socio-relational influences on decisions, such as a news clip on a social media platform, a friend’s comment or suggestion, a notification from an app, and so on. These kinds of cases allow us to track attitudes about the relationship between social embeddedness and autonomous decision-making. Our studies examine how this putative tension, between our fundamental sociality and the ideal of personal autonomy, is negotiated by people with respect to their day-to-day interactions. In line with empirical studies that have explored how the public perceives of beliefs in free will and agency (Shepard and Reuter, 2012; Nadelhoffer et al., 2014) and the influence that such beliefs may have upon real world behaviors (Shariff et al., 2014; Protzko et al., 2016; Martin et al., 2017), we anticipate that our studies of perceptions of undue influence will open new avenues of inquiry into the “folk conception” of autonomy.

Our study focuses on two aspects of influences on decisions. The first examines the *rationality status* of the influence, a classic condition for accounts of autonomy. Philosophically, it is generally accepted that a person needs to have both adequate capacity and opportunity to evaluate and endorse the reasons for making a particular decision vis-à-vis her conception of her desires and wants (Buss and Westlund, 2018). The psychological and neuroscientific evidence, by contrast, has shown that many of the cognitive processes that result in action are less-than-rational, as classically conceived (Felsen and Reiner, 2011). But how are public attitudes of the undueness of influences affected by rationality status? We empirically tested the hypothesis that RATIONAL influences (operationally defined as those that explicitly offer reasons) are perceived as less undue than ARATIONAL influences (defined as those that fail to explicitly offer any reasons) in the context of everyday decision-making.

The second aspect concerns the relationship between the influencer and the decision-maker. It has already been well-established that our decisions are more readily influenced by agents with whom we (know we) share a worldview than by those with whom we do not (Cohen, 2003; Kahan and Braman, 2006; Kahan, 2013). We have proposed that, at least in some cases, this results from our “preauthorizing” the former agents (Niker et al., 2016). What is not yet known, and what we test here, is whether perceptions of the undueness of an influence depend on whether the influencer shares one’s worldview. Thus, we tested the hypothesis that attitudes about influences depend on *preauthorization status*. Specifically, we predicted that an influence from a PREAUTHORIZED agent, who is known to share one’s worldview, would be perceived as less undue than the identical influence from a NON-PREAUTHORIZED agent, who is not.

We addressed these questions using contrastive vignettes that systematically varied both *rationality* and *preauthorization statuses*. In support of our hypotheses, we found that the influence was perceived to be less undue when it was RATIONAL and when it originated from a PREAUTHORIZED agent. The relative magnitudes of the effects of *rationality* and *preauthorization statuses* on the perceived undueness of an influence, and interactions between these factors, depended on the context of the decision. A follow-up study demonstrated that the effect of preauthorization depended on having a shared worldview with the agent, and so could not be explained solely by familiarity. We suggest that these results support an understanding of preauthorization as an evaluative stance by which an actor gives a certain agent preferential access to influencing her decisions. Specifically, the agent’s influence is incorporated in relevant future interactions *without* needing to be consciously evaluated, and without impacting the actor’s perception of the control she has over the resultant decision (Niker et al., 2016).

After detailing the methodology and results of our studies in the next two sections, we describe in the Discussion how our results inform the folk conception of autonomy, that is, the set of internal accounts held by the public when they consider the concept of autonomy (Stich and Ravenscroft, 1994). We also explore the implications of these results for a range of real-world debates, such as those over the ethics of public policies designed to influence decisions (i.e., “nudges”) and information sharing in social networks.

## Materials and Methods

**FIGURE 1:**
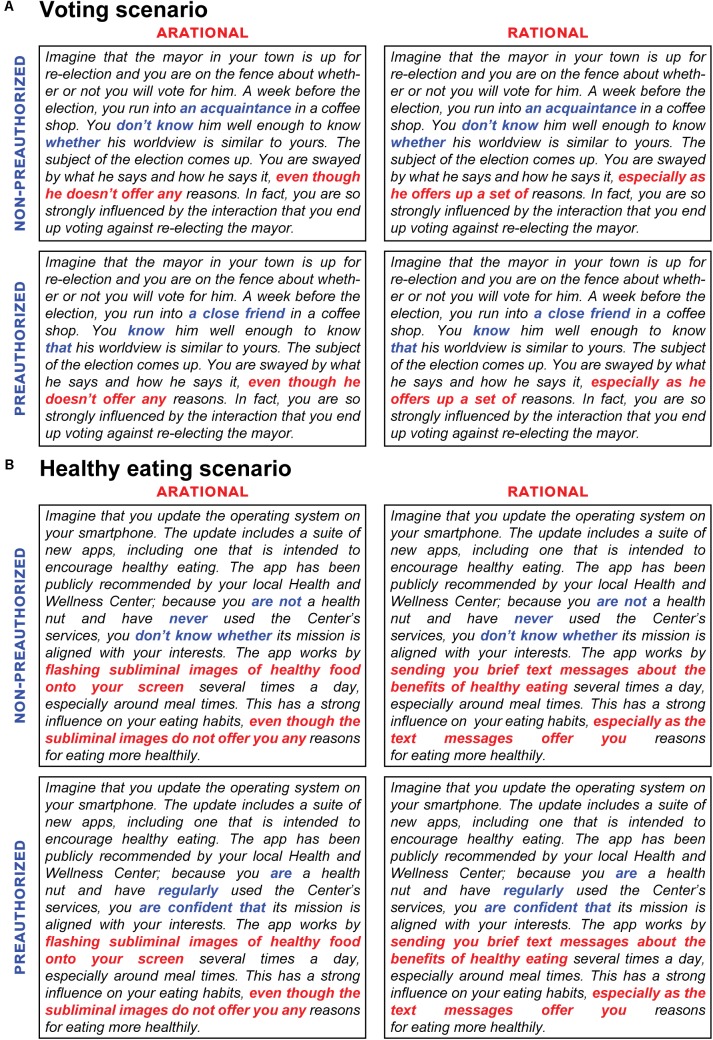
Contrastive vignettes. **(A)** The four vignettes used in the voting scenario. All differences between the ARATIONAL (left) and RATIONAL (right) conditions are shown in red; all differences between the NON-PREAUTHORIZED (upper) and PREAUTHORIZED (lower) conditions are shown in blue. Since questions are identical for all respondents, any differences in responses between conditions must be due to the differences between vignettes (shown in red and blue). Black text is identical across all conditions. **(B)** As in **(A)**, for the healthy eating scenario.

Respondents from the United States were recruited via Amazon’s Mechanical Turk. As with other studies using Mechanical Turk, our sample of respondents was representative of, though somewhat younger and better educated than, the general United States population (Paul, 2016; **Table 1**). Following acceptance of informed consent, each respondent was randomly assigned to read one (and only one) of several contrastive vignettes (Burstin et al., 1980) that asked the respondent to imagine a scenario (requiring a decision about either voting or healthy eating) in which an external influence affected their decision (**Figure 1**; see **Supplementary Figure S1**). The *rationality status* of the type of influence and the *preauthorization status* of the source of the influence varied across vignettes while all other aspects of the vignettes were identical; *rationality status* was either RATIONAL or ARATIONAL, and *preauthorization status* was either PREAUTHORIZED or NON-PREAUTHORIZED. The respondents then answered a set of questions designed to capture their attitudes about the extent to which the influence was undue (“To what extent would you find this influence objectionable?,” “To what extent would you find this influence manipulative?,” and “To what extent would you find this influence welcome?”), quantified on a scale of 1 (“Not at all”) to 100 (“Very much so”). Responses to each question were highly correlated (voting scenario: Objectionable vs. Manipulative: *r* = 0.62, *p* < 10^-44^; Objectionable vs. Welcome: *r* = -0.62, *p* < 10^-44^; Manipulative vs. Welcome: *r* = -0.55, *p* < 10^-32^; healthy eating scenario: Objectionable vs. Manipulative: *r* = 0.55, *p* < 10^-34^; Objectionable vs. Welcome: *r* = -0.68, *p* < 10^-59^; Manipulative vs. Welcome: *r* = -0.49, *p* < 10^-26^), confirming that these questions functioned complementarily to capture perceptions of a unitary concept that we refer to as “undueness.” Since the only differences between vignettes were the *rationality status* and *preauthorization status*, any differences between groups (e.g., the RATIONAL and ARATIONAL groups) in responses to these questions must be due to the differences between the vignettes along one of these dimensions (e.g., *rationality status*; differences shown in red in **Figure 1**).

**Table 1:** Demographic characteristics of respondents.

Demographic factor	Distribution of respondents
Age	Range: 18–72
	Median: 33
	Mean ± SD: 35.7 ± 11.3
Biological sex	Female: 50.8%
	Male: 49.2%
Highest education level	Some high school: 0.6%
	High school diploma: 9.5%
	Some college or university: 31.0%
	College or university degree: 41.5%
	Some post-graduate: 4.2%
	Post-graduate degree: 13.2%
Annual household income:	<$22,500: 17.0%
	$22,500–39,999: 22.6%
	$40,000–59,999: 19.4%
	$60,000–89,999: 21.4%
	$90,000 or more: 17.6%
	Prefer not to say: 2.1%

Following these three primary outcome measures, we asked respondents two questions that allowed us to assess whether their perception matched the conditions to which they were assigned [*rationality status* check: “He offered reasons for why you should vote against re-electing the mayor” (voting scenario); “The app provided you with reasons for eating more healthily” (healthy eating scenario); *preauthorization status* check: “You are confident that he shares your worldview” (voting scenario); “You are confident that the mission of the Health and Wellness Center is aligned with your interests” (healthy eating scenario)], quantified on a scale of 1 (“Strongly disagree”) to 100 (“Strongly agree”). In addition, after asking respondents “To what extent would you find this influence objectionable?,” we asked them to “Please tell us why you answered as you did.” We qualitatively assessed the responses to this question as an additional check to further ensure that the respondents understood the vignette, their assigned condition, and the questions. We also used these responses, in a previous pilot version of our study, to fine-tune our vignettes and questions in the present study. Finally, respondents in the voting scenario were asked, “To what extent is voting in a mayoral election important to you?,” quantified on a scale of 1 (“Not very important”) to 100 (“Very important”) (mean ± SD: 69.5 ± 25.5). We used this response to determine whether attitudes about influences on voting decisions depended on whether voting in a mayoral election was considered to be important. We found that they did not; our overall results (described below) were unchanged when data from respondents for whom voting in a mayoral election was not very important (≤50) were excluded.

A follow-up study replicated the above procedure in order to examine two potential components of preauthorization by independently varying *familiarity status* (FAMILIAR vs. UNFAMILIAR) and *worldview status* (SHARED WORLDVIEW vs. UNKNOWN WORLDVIEW) in a modified version of the voting scenario.

We then asked respondents to complete a short form that allowed us to place each respondent on a communitarian-individualist scale (Kahan, 2012). Finally, we verified comprehension of the vignettes by asking respondents to identify the topic of the vignette from a multiple-choice list; 10 respondents with incorrect responses were excluded from subsequent analysis. Respondents were also excluded for taking too little (<2 min) or too much (>15 min) time to complete the experiment (158 respondents excluded). All respondents were compensated $0.40 for completion of the survey. The entire set of screens presented to the respondents is provided (**Supplementary Figure S1**), and all data included in our analyses are available as a supplementary dataset (**Supplementary Data Sheet S1**).

All analyses were performed in MATLAB; primary built-in functions used were ttest2, corrcoef, and fitlm. All experiments were approved by The University of British Columbia Behavioral Research Ethics Board. All subjects gave written informed consent in accordance with the Declaration of Helsinki.

## Results

We used contrastive vignettes (Burstin et al., 1980; Felsen et al., 2013) to examine attitudes about how the type and source of external influences on decisions affect people’s perceptions about the undueness of those influences. Each respondent was presented with a single vignette asking them to imagine a scenario in which an influence affects an everyday decision of theirs (see section “Materials and Methods”; **Figure 1**). In the voting scenario, the respondent’s vote in a mayoral election is influenced by another person (**Figure 1A**), while in the healthy eating scenario, the respondent’s decision to eat more healthily is influenced by an app on their smartphone (**Figure 1B**). In both scenarios, we systematically varied the context in which the decision is influenced along two independent dimensions: *rationality status* (RATIONAL or ARATIONAL; **Figure 1**, red) and *preauthorization status* (PREAUTHORIZED or NON-PREAUTHORIZED; **Figure 1**, blue) (see section “Materials and Methods”). We verified that respondents’ perception matched the conditions to which they were assigned based on their responses to the perception check questions. As expected, responses to the *rationality status* check differed between the RATIONAL and ARATIONAL conditions (voting scenario, *p* < 10^-106^; healthy eating scenario, *p* < 10^-84^, two-tailed unpaired *t*-tests) and responses to the *preauthorization status* check differed between the PREAUTHORIZED and NON-PREAUTHORIZED conditions (voting scenario, *p* < 10^-73^; healthy eating scenario, *p* < 10^-29^, two-tailed unpaired *t*-tests), confirming the intended contrasts between our vignettes. Nevertheless, to ensure interpretability of our results, respondents with mismatched perceptions (ARATIONAL, >50; RATIONAL, ≤50; NON-PREAUTHORIZED, >50; PREAUTHORIZED, ≤50) were excluded from subsequent analysis, resulting in 403 respondents included in the voting scenario and 428 respondents included in the healthy eating scenario [although not excluding respondents with mismatched perceptions revealed that perceived condition predicted attitudes similarly to assigned condition (**Table 2**, rows 4–5)]. The questions that followed each of these vignettes examined the degree to which the influence was perceived as undue (see section “Materials and Methods”).

**Table 2:** Logistic regression results.

	Predictor	How Objectionable?	How Manipulative?	How Welcome?
1	*Rationality status*	V: β = –15 (*p* < 10^-22^)	V: β = –9.1 (*p* < 10^-9^)	V: β = 13 (*p* < 10^-22^)
		H: β = –7.2 (*p* < 10^-5^)	H: β = –11 (*p* < 10^-10^)	H: β = 4.0 (*p* < 0.01)
2	*Preauthorization status*	V: β = –7.1 (*p* < 10^-5^)	V: β = –7.7 (*p* < 10^-6^)	V: β = 8.0 (*p* < 10^-9^)
		H: β = –11 (*p* < 10^-11^)	H: β = –8.9 (*p* < 10^-7^)	H: β = 14 (*p* < 10^-19^)
3	*Rationality status* ×*Preauthorization status*	V: β = 1.4 (*p* = 0.31)	V: β = 1.2 (*p* = 0.39)	V: β = 0.28 (*p* = 0.82)
		H: β = 0.88 (*p* = 0.57)	H: β = –3.9 (*p* < 0.05)	H: β = 2.9 (*p* = 0.055)
4	Perceived *rationality status*	V: β = –0.37 (*p* < 10^-10^)	V: β = –0.19 (*p* < 10^-3^)	V: β = 0.27 (*p* < 10^-8^)
		H: β = –0.23 (*p* < 10^-4^)	H: β = –0.15 (*p* < 0.01)	H: β = 0.02 (*p* = 0.59)
5	Perceived *preauthorization status*	V: β = –0.26 (*p* < 10^-5^)	V: β = –0.20 (*p* < 10^-3^)	V: β = 0.28 (*p* < 10^-7^)
		H: β = –0.46 (*p* < 10^-15^)	H: β = –0.22 (*p* < 10^-4^)	H: β = 0.41 (*p* < 10^-14^)
6	*Preauthorization status* × Communitarian-Individualist score	V: β = –5.7 (*p* = 0.090)	V: β = –0.70 (*p* = 0.83)	V: β = –3.2 (*p* = 0.26)
		H: β = –3.1 (*p* = 0.41)	H: β = –0.42 (*p* = 0.91)	H: β = –3.2 (*p* = 0.38)
7	*Preauthorization status* × Sex	V: β = 0.011 (*p* = 0.99)	V: β = 0.79 (*p* = 0.59)	V: β = –0.45 (*p* = 0.72)
		H: β = 0.49 (*p* = 0.75)	H: β = 0.69 (*p* = 0.66)	H: β = –0.70 (*p* = 0.64)
8	*Familiarity status*	β = –4.0 (*p* < 0.01)	β = –2.7 (*p* = 0.056)	β = 3.2 (*p* < 0.05)
9	*Shared worldview status*	β = –7.5 (*p* < 10^-6^)	β = –6.3 (*p* < 10^-5^)	β = 8.9 (*p* < 10^-9^)
10	Perceived *familiarity status*	β = –0.068 (*p* = 0.29)	β = –0.094 (*p* = 0.13)	β = 0.11 (*p* = 0.055)
11	Perceived s*hared worldview status*	β = –0.24 (*p* < 10^-4^)	β = –0.27 (*p* < 10^-4^)	β = 0.35 (*p* < 10^-9^)

*Rows 1–3: responses to each question were regressed against *rationality status* (*ARATIONAL* = –1; *RATIONAL* = 1; row 1), *preauthorization status* (*NON-PREAUTHORIZED* = –1; *PREAUTHORIZED* = 1; row 2), and their interaction (row 3). *V*, *VOTING* scenario; *H*, *HEALTHY EATING* scenario. Rows 4–5: as in rows 1–2, but with responses to perception check questions (ranging from 1 to 100) instead of assigned conditions as predictors. For this analysis, no respondents were excluded for a mismatch between their perceived and assigned conditions. Row 6: as in rows 1–3 without *rationality status × preauthorization status* interaction, and with the communitarian-individualist score [rescaled from –1 (maximally communitarian) to 1 (maximally individualist)] and *preauthorization status*× communitarian-individualist interaction as additional predictors. Row 7: as in rows 1–3 without *rationality status × preauthorization status* interaction, and with Sex (Female = –1; Male = 1) and *preauthorization status*× Sex interaction as additional predictors. Rows 8–9: responses to each question were regressed against *familiarity status* (*UNFAMILIAR* = –1; *FAMILIAR* = 1; row 8) and *worldview status* (*UNKNOWN WORLDVIEW* = –1; *SHARED WORLDVIEW* = 1; row 9). Rows 10–11: as in rows 8–9, but with responses to perception check questions (ranging from 1 to 100) instead of assigned conditions as predictors. For this analysis, no respondents were excluded for a mismatch between their perceived and assigned conditions. The black and gray (corresponding to significant and nonsignificant weights) in the table seems to have been lost. For this analysis, no respondents were excluded for a mismatch between their perceived and assigned conditions. For all rows, significant weights (at p < 0.05) are shown in black and non-significant weights are shown in gray.*

**FIGURE 2:**
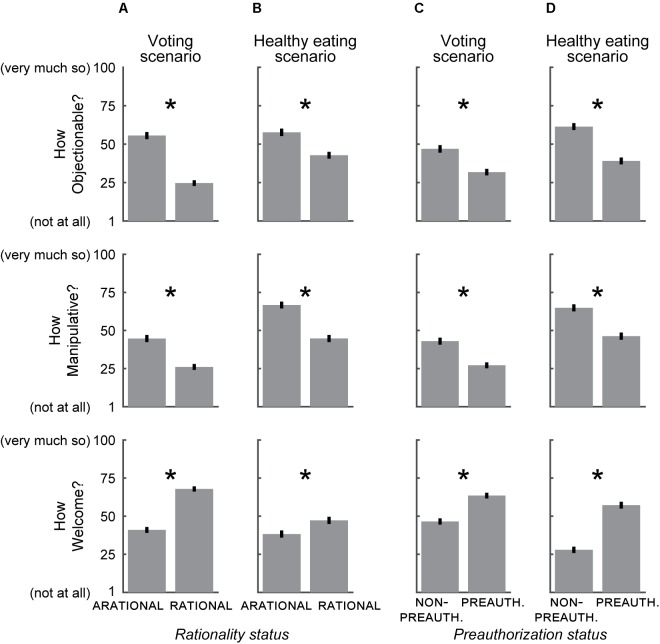
Effect of *rationality status* and *preauthorization status* on the extent to which an influence was perceived as undue. **(A)** In the voting scenario, an ARATIONAL influence was perceived as more Objectionable (upper), more Manipulative (middle) and less Welcome (lower) than a RATIONAL influence. **(B)** As in **(A)**, for the healthy eating scenario. **(C)** In the voting scenario, an influence from a NON-PREAUTHORIZED source was perceived as more Objectionable (upper), more Manipulative (middle), and less Welcome (lower) than an influence from a PREAUTHORIZED source. **(D)** As in **(C)**, for the healthy eating scenario. Error bars ± SEM. ^∗^*p* < 0.01, two-tailed unpaired *t*-test.

**FIGURE 3:**
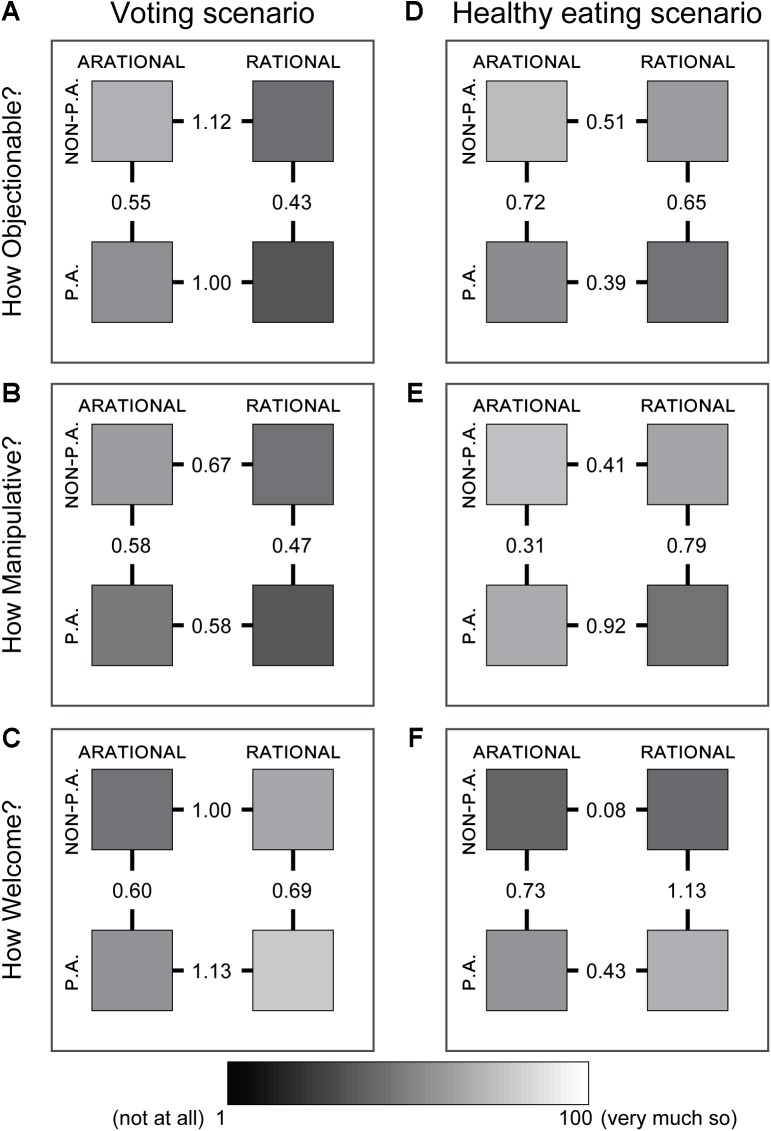
Effect sizes between and across *rationality status* and *preauthorization status* conditions. **(A)** Effect sizes (Cohen’s d) between groups’ (squares) perceptions of the extent to which the influence was perceived as Objectionable in the voting scenario. Shading corresponds to mean group response. NON-P.A., NON-PREAUTHORIZED; P.A., PREAUTHORIZED. **(B,C)** As in **A**, for the extent to which the influence was perceived as Manipulative **(B)** and Welcome **(C)**. **(D–F)** As in **(A–C)**, for the healthy eating scenario.

Given the importance of the opportunity for critical reflection for philosophical accounts of autonomy, we expected that ARATIONAL influences would be perceived as more undue than RATIONAL influences. In the voting scenario, the ARATIONAL influence was indicated by describing how a person sways the respondent not to vote for the mayor in an upcoming election “even though he doesn’t offer any reasons” (**Figure 1A**, left boxes, red), while the RATIONAL influence was indicated by describing how the respondent is identically swayed by a person “offer[ing] up a set of reasons” (**Figure 1A**, right boxes, red). As expected, the respondents in the RATIONAL condition considered the influence to be less Objectionable (*p* < 10^-22^, two-tailed unpaired *t*-test), less Manipulative (*p* < 10^-8^, two-tailed unpaired *t*-test), and more Welcome (*p* < 10^-21^, two-tailed unpaired *t*-test) than respondents in the ARATIONAL condition (**Figure 2A**; **Figures 3A–C** shows effect sizes between each group of respondents; **Table 2**, row 1 shows regression weights). In the healthy eating scenario, an app was described as having a strong influence on eating habits, and works either by flashing subliminal images of healthy food that “do not offer you any reasons for eating more healthily” (ARATIONAL; **Figure 1B**, left boxes, red) or by sending text messages several times a day that offer “reasons for eating more healthily” (RATIONAL; **Figure 1B**, right boxes, red). As in the voting scenario, respondents in the RATIONAL condition again considered the influence to be less Objectionable, less Manipulative, and more Welcome than respondents in the ARATIONAL condition (Objectionable: *p* < 10^-5^; Manipulative: *p* < 10^-10^; Welcome: *p* < 0.01, two-tailed unpaired *t*-tests; **Figures 2B**, **3D–F** and **Table 2**, row 1). These data support the idea that people generally perceive influences that are RATIONAL as less undue, and therefore less of an infringement on personal autonomy, than influences that are ARATIONAL.

We next examined how attitudes were affected by *preauthorization status*. In the NON-PREAUTHORIZED condition of the voting scenario, the source of the influence was described as an acquaintance whose worldview is unknown (**Figure 1A**, upper boxes, blue), while in the PREAUTHORIZED condition, the source of the influence was described as a close friend whose worldview is aligned with that of the respondent (**Figure 1A**, lower boxes, blue). We found that respondents perceived influences from PREAUTHORIZED sources as less Objectionable, less Manipulative, and more Welcome than otherwise-identical influences from NON-PREAUTHORIZED sources (Objectionable: *p* < 10^-5^; Manipulative: *p* < 10^-6^; Welcome: *p* < 10^-8^, two-tailed unpaired *t*-tests; **Figures 2C**, **3A–C** and **Table 2**, row 2). For all questions, the effects of *preauthorization status* were independent of the effects of *rationality status* (**Table 2**, row 3). In the healthy eating scenario, the source of the influence was a smartphone app recommended by the respondent’s local Health and Wellness Center. In the PREAUTHORIZED condition, the respondent is described as a “health nut” who regularly uses the Center’s services and is therefore confident that its mission is aligned with their interests (**Figure 1B**, upper boxes, blue). In contrast, in the NON-PREAUTHORIZED condition, the respondent is described as having never used the Center’s services, and who therefore does not know whether its mission is aligned with their interests (**Figure 1B**, lower boxes, blue). Respondents again deemed the influence from the PREAUTHORIZED source as less Objectionable, less Manipulative, and more Welcome than the same influence from the NON-PREAUTHORIZED source (Objectionable: *p* < 10^-11^; Manipulative: *p* < 10^-7^; Welcome: *p* < 10^-20^, two-tailed unpaired *t*-tests; **Figures 2D**, **3D–F** and **Table 2**, row 2). In this scenario, we found that the extent to which the influence was perceived to be Manipulative was affected by an interaction between *rationality status* and *preauthorization status*, and there was a trend toward an interaction in response to the Welcome question, while there was no interaction in response to the Objectionable question (**Table 2**, row 3).

In order to examine the relative strength of *rationality status* and *preauthorization status* on the perceived undueness of an influence, we visualized the mean responses of all four groups (2 dimensions × 2 conditions) as well as the effect sizes between pairs of groups (**Figure 3**). Consistent with the results of our regression analysis (**Table 2**, rows 1–2), we found that *rationality status* had a somewhat stronger effect in the voting scenario, whereas *preauthorization status* had a somewhat stronger effect in the healthy eating scenario. In the voting scenario, we found that an ARATIONAL influence from a PREAUTHORIZED source was perceived as somewhat more undue than a RATIONAL influence from a NON-PREAUTHORIZED source, and that the effect sizes between ARATIONAL and RATIONAL groups were generally larger than those between NON-PREAUTHORIZED and PREAUTHORIZED groups (**Figures 3A–C**). In contrast, in the healthy eating scenario we found that an ARATIONAL influence from a PREAUTHORIZED source was perceived as somewhat less Objectionable and more Welcome than a RATIONAL influence from a NON-PREAUTHORIZED source, and that the effect sizes between NON-PREAUTHORIZED and PREAUTHORIZED groups were generally larger than those between ARATIONAL and RATIONAL groups for these questions [**Figures 3D,F**; this pattern did not hold for perceptions of how Manipulative the influence was (**Figure 3E**), although perceived manipulation was affected by an interaction between *rationality status* and *preauthorization status* (**Table 2**, row 3)]. Thus, while both *rationality status* and *preauthorization status* affect the perceived undueness of an influence in each scenario (**Figure 2** and **Table 2**, rows 1–2), the relative extent to which they do so, and whether they interact, may be situationally dependent.

After answering our primary questions and perception checks, respondents were also asked to complete a scale that maps respondents onto a continuum from “communitarian” to “individualist” (Kahan, 2012; see section “Materials and Methods”). We reasoned that more communitarian respondents might be more sensitive to *preauthorization status*, i.e., they would be less likely than individualistic respondents to perceive an influence from a PREAUTHORIZED source as undue. Surprisingly, we found that this was not the case: the effect of *preauthorization status* was not modulated by score on the communitarian-individualist scale (**Table 2**, row 6). We also found that men were no more likely than women to perceive an influence from a PREAUTHORIZED source as undue (**Table 2**, row 7).

These results support our prediction that perceptions of otherwise-identical influences are context dependent: RATIONAL influences are perceived as less undue than those that are ARATIONAL (**Figures 2A,B**), consistent with earlier findings that showed that the public generally prefers overt influences rather than covert influences (Felsen et al., 2013; Sunstein, 2016a). In addition, an influence is considered less undue when it is exerted by a PREAUTHORIZED than a NON-PREAUTHORIZED source, irrespective of whether the source is an interpersonal or institutional agent (**Figures 2C,D**). However, our use of preauthorization thus far incorporates several related concepts, notably familiarity (acquaintance or close friend) and worldview (unknown or shared), which covaried between the NON-PREAUTHORIZED and PREAUTHORIZED conditions. To what extent are our findings (**Figures 2**, **3**) due to each of these components of preauthorization?

In order to dissociate the effects of these components, we performed another set of experiments using a variant of the voting scenario in which we systematically varied the context in which the decision is influenced in the two independent dimensions of *familiarity status* (FAMILIAR or UNFAMILIAR) and *worldview status* (SHARED WORLDVIEW or UNKNOWN WORLDVIEW). We examined the degree to which the influence was perceived as undue via the same questions as above (i.e., how Objectionable, Manipulative, and Welcome; see section “Materials and Methods”). We again used perception checks to verify that respondents’ perception matched the conditions to which they were assigned [responses to the *familiarity status* check differed between the FAMILIAR and UNFAMILIAR conditions (*p* < 10^-73^, two-tailed unpaired *t*-test); responses to the *worldview status* check differed between the SHARED WORLDVIEW and UNKNOWN WORLDVIEW conditions (*p* < 10^-90^, two-tailed unpaired *t*-test)]. As above, respondents with mismatched perception (UNFAMILIAR, >50; FAMILIAR, ≤50; UNKNOWN WORLDVIEW, >50; SHARED WORLDVIEW, ≤50) were excluded from subsequent analysis, resulting in 382 respondents [although not excluding such respondents again revealed that perceived condition predicted attitudes similarly to assigned condition (**Table 2**, rows 10–11)].

**FIGURE 4:**
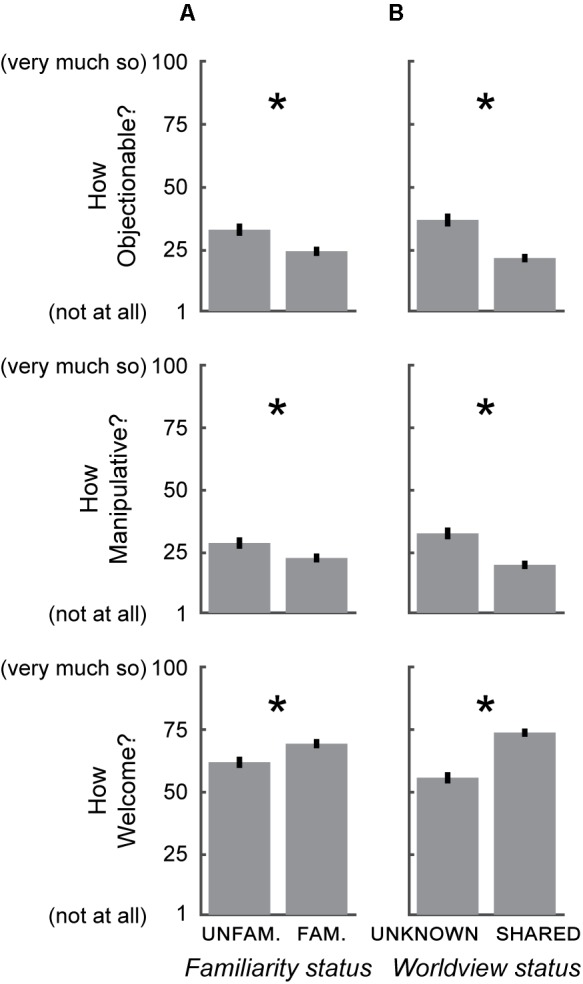
Effect of *familiarity status* and *worldview status* on the extent to which an influence was perceived as undue. **(A)** An influence from an UNFAMILIAR source was perceived as more Objectionable (upper), more Manipulative (middle), and less Welcome (lower) than an influence from a FAMILIAR source. **(B)** An influence from a source with an UNKNOWN WORLDVIEW was perceived as more Objectionable (upper), more Manipulative (middle) and less Welcome (lower) than an influence from a source with a SHARED WORLDVIEW. Error bars ± SEM. ^∗^*p* < 0.01, two-tailed unpaired *t*-test.

We found that the respondents in the FAMILIAR condition considered the influence to be less Objectionable, less Manipulative, and more Welcome than respondents in the UNFAMILIAR condition (Objectionable: *p* < 0.01; Manipulative: *p* < 0.05; Welcome: *p* < 0.01, two-tailed unpaired *t*-tests; **Figure 4A** and **Table 2**, row 8). Likewise, respondents in the SHARED WORLDVIEW condition considered the influence to be less undue than respondents in the UNKNOWN WORLDVIEW condition (Objectionable: *p* < 10^-6^; Manipulative: *p* < 10^-5^; Welcome: *p* < 10^-9^, two-tailed unpaired *t*-tests; **Figure 4B** and **Table 2**, row 9). These results suggest that familiarity and worldview are both components of preauthorization, and that worldview has at least as strong an effect as familiarity on attitudes about how influences on decisions affect autonomy (compare magnitudes of regression results between rows 8 and 9, and between rows 10 and 11).

## Discussion

We provide evidence that perceptions of the undueness of influences on decisions are context-dependent. When respondents were presented with otherwise-identical influences, they perceived those that were RATIONAL to be less undue than those that were ARATIONAL (**Figures 2A,B**) and those that were exerted by a PREAUTHORIZED source as less undue than those that derived from a NON-PREAUTHORIZED source (**Figures 2C,D**). The preauthorization effect was driven both by familiarity with the source of the influence (**Figure 4A**), as well as by shared worldview (**Figure 4B**), with the latter having at least as strong of an effect. There are likely additional factors that drive public attitudes about influences on decision-making, and these represent fertile ground for future experimental investigation. In addition, the attitudes that we examined here are likely to vary across cultures, particularly between those with divergent views on the centrality of personal autonomy; these cultural variations can also be examined in future studies. To our knowledge, however, our data are the first to describe how people perceive the distinction between those influences that are appropriate and those that are undue in the context of everyday decision-making, and thus are an important initial step in providing insight into the folk conception of autonomy.

In particular, these observations provide novel insights into the phenomenology of agency, by exploring the degree to which people perceive that they retain control over their decision-making while navigating the real world in which decisions will often be influenced by external agents. Without explicitly querying public sentiments about free will, these experiments shed light upon the degree to which people view autonomous decisions as being caused entirely of their own accord, and the conditions under which outside influences may be integrated into one’s decision-making while still preserving perceived autonomy, an issue that we expand upon below.

Recent debate among theorists of autonomy has centered on the tension between our social ties and our ability to make our own decisions. While the debate in academic circles has been searching for a nuanced answer to how to understand which social and relational influences support the development and exercise of autonomy and which others hinder it (Dworkin, 1988; Friedman, 2003), popular culture has continued to proliferate an overly individualistic view of autonomy. Our finding that, regardless of how individualistic they identified as (on the communitarian-individualist scale), people perceive an influence from a PREAUTHORIZED agent as less undue than the identical influence from a NON-PREAUTHORIZED agent is all the more interesting in light of this persistent cultural glorification of the rugged individual making decisions “for himself, by himself” (Callero, 2013; Brownlee, 2017). Our data suggest that people tend to see through this “myth of individualism” (Code, 2016) when considering how they navigate their own lives; instead, they appear to ascribe to a more *relational* conception of autonomy, which understands autonomous decision-making as partly conditional on the cultivation of the types of relationships and interpersonal contexts that can support its realization (Mackenzie and Stoljar, 2000; Specker Sullivan and Niker, 2018).

We suggest, then, that the folk conception of autonomy aligns with recent trends in the philosophical debate toward social and relational conceptions of autonomy. The data suggest that people experience influences from those they have preauthorized (in this domain) as something that does not negatively affect the control that they perceive themselves to have over their decision-making processes. In certain situations (such as in our healthy eating scenario), arational influences from a preauthorized source are perceived as less objectionable and more welcome than a rational influence from a non-preauthorized source. This finding highlights the relationship between conscious cognitive processes and control over one’s behavior in a way that supports our theoretical conception of preauthorization as “a process by which an individual gives a certain agent preferential access to influencing her decision-making processes” (Niker et al., 2016, p. 27). Whether we do so explicitly or as a form of tacit knowledge (Cianciolo et al., 2006), we suggest that *the act of preauthorization* represents an adaptive cognitive process: the brain evaluates external agents and classifies them by how reliable their information may be. Once “tagged” in this way, it is plausible that the skeptical filter that we apply to information deriving from preauthorized sources is relaxed, making it easier for these sources to influence our decision-making.

Our data are also relevant for applied debates, such as the public policy debate over the ethics of using “nudges” to modify citizens’ behavior (Hausman and Welch, 2010; Grune-Yanoff, 2012; Niker, 2018). Nudges are small changes to the environment, or “choice architecture,” designed to promote particular welfare-promoting choices without coercively limiting the range of options (Thaler and Sunstein, 2008). Although data on public attitudes alone cannot resolve the relevant ethical questions, the debate over the permissibility of nudging should at least be informed by whether, and to what extent, people perceive nudges to be undue influences on their decision making (Sunstein, 2016b). Previous studies have shown that people prefer overt to covert nudges (Felsen et al., 2013), as well as nudges that appeal to “System 2” rather than “System 1” thinking (Jung and Mellers, 2016; Sunstein, 2016a) – in both cases because the former are considered less manipulative. Consistent with these observations, our data demonstrate that people perceive RATIONAL influences as less undue than ARATIONAL influences, whether they are in the form of nudges (healthy eating scenario) or advice (voting scenario). Together, these studies strongly suggest that nudges that at least allow individuals to enlist cognitive resources consistent with reasoning before making a decision are perceived of as less undue than those that influence decision-making in a less transparent fashion.

More distinctively, our results suggest that the relationship between the nudger and nudged is also likely to play an important role in determining attitudes about the acceptability of behavior-modification interventions. We suggest that preauthorization – not simply familiarity but having a shared worldview with the influencing agent – is one of the factors explaining why certain nudges may be perceived as more or less welcome depending on which agent deploys them. Consequently, our results may partially explain the phenomenon of *partisan nudge bias*, whereby attitudes toward particular policy goals or policymakers – i.e., whether they align with the actor’s goals and commitments – affect attitudes about the acceptability of the policy itself (Tannenbaum et al., 2017).

Additionally, actors may not only preauthorize but also *anti-preauthorize* certain agents. For example, individuals who are committed to getting their news from one (partisan) news source might actively ignore information from another (opposingly partisan) news source, perhaps because the information may be perceived as unduly influencing their beliefs. Although we did not explicitly test this hypothesis, the results of two recent surveys support this idea (Media Insight Project, 2017; Silverman, 2017). Indeed, in concert with confirmation bias (Nickerson, 1998), preauthorization may readily lead to the development of so-called “echo chambers” in which we lend added credence to information that derives from those with whom we share a worldview while markedly discounting information from those who we have flagged as having opposing viewpoints (Bessi et al., 2016; Del Vicario et al., 2016; Editorial, 2017).

Such developments have real-world implications for democracy in the age of social media, including the ethics of the practice of “moral reframing,” in which political arguments are framed to appeal to the values of those targeted for persuasion (Feinberg and Willer, 2015), and the problems raised by algorithmically-curated “filter bubbles.” These approaches are effective at influencing decisions, and have been the subject of recent debates over the power social media to shape public opinion about critical topics like political campaigns. Clarifying how these influences are perceived can help guide the development of responsible online choice architecture.

## Conclusion

We have shown that external influences are perceived as more or less undue depending upon socio-relational context. Our empirical results about the folk conception of autonomy align with well-developed philosophical arguments and provide evidence that a new construct, preauthorization, plays a role in the delicate dance between influencer and decision maker.

## Data Availability

The datasets generated by this study are provided as supplementary material (**Supplementary Data Sheet S1**).

## Author Contributions

FN, PR, and GF conceived and designed the study, collected and analyzed the data, and wrote the manuscript.

## Conflict of Interest Statement

The authors declare that the research was conducted in the absence of any commercial or financial relationships that could be construed as a potential conflict of interest.

## Supplementary Material

FIGURE S1 Full set of screens presented to the respondents.

DATA SHEET S1 Excel file containing all data included in this study.
