# Supplementary material for: Perceptions of Undue Influence Shed Light on the Folk Conception of Autonomy
Source: Front Psychol. 2018 Aug 8;9:1400. doi: 10.3389/fpsyg.2018.01400 (PMC6092627; doi:10.3389/fpsyg.2018.01400)
Supplement: FIGURE S1 — Full set of screens presented to the respondents. [file Image_1.PDF]

The pages that follow contain screenshots of the full set of vignettes and questions that were seen by respondents. Each respondent read one and only one of the vignettes and then all were presented with the common questions.

Instructions

Instructions: This research is important to us, and we would appreciate your full cooperation.

Please complete this study in one session without taking breaks. (This study will take less than 5 minutes to complete.) At the top of each page, you will find a short scenario, followed by some questions. The scenario will be repeated at the top of each page, but the questions will change.

Before you begin the study, we ask that you:

Turn off any music and television in your immediate surroundings.
Do not use your cell phone.
Do not use other web browser window/tabs.

Right now, do you have at least 5 minutes of uninterrupted time in which you can complete this survey?

○ Yes ○ No

Do you agree to complete this survey in one sitting, without taking any breaks, and without talking to anyone else?

○ Yes ○ No

Have you turned off any phones, televisions, music, and other media devices in your immediate surroundings?

○ Yes ○ No

If you have answered "No" to any of the questions above, please return to the survey when you are able to fulfill all of the requirements for participation.

If you have answered "Yes" to all of the questions above, please click the "Next" button to proceed with the survey.

Next

Informed Consent

Informed Consent

Principal Investigator: Peter B. Reiner, Professor, National Core for Neuroethics, University of British Columbia, 604.827.5836.

Sponsor: None.

Study Aim: To explore social attitudes.

Study Procedures: You will be randomly assigned to complete a short online questionnaire; the entire study should take less than 5 minutes. You will be asked to read several hypothetical scenarios.

Confidentiality: All survey responses will be kept anonymous. Only the principal investigator and research assistants will have access to the data; computer files will be password protected.

Incentive: A payment of US $0.40 for completion of the survey.

Voluntary Participation: Please note that your participation is entirely voluntary; however, submitting the survey expresses your consent to participate and the use of the information you provide for the purpose of our study. You may withdraw from the survey at any point prior to submission by simply closing your browser window.

Conflict of Interest: The authors of this study are not affiliated with any pharmaceutical company or commercial enterprise and declare absolutely no conflict of interest.

Contact for more Information: If you have any questions or would like further information about this study, you may contact the Principal Investigator, Peter Reiner at peter.reiner@ubc.ca.

Contact for Concerns about the Rights of Research Participants: If you have any concerns about your treatment or rights as a research participant, you may contact the Research Subject Information Line in the UBC Office of Research Services at 604.822.8598 or email RSIL@ors.ubc.ca

- [ ] I consent to participate
- [ ] I do not consent to participate

Next

After reading and completing the informed consent, participants are randomly assigned to read one of the following eight vignettes. After reading their particular vignette (and answering the questions on that page of the survey), they are directed to the ‘Common Questions’ and the remainder of the survey.

Voting scenario, PREAUTHORIZED, ARATIONAL

Imagine that the mayor in your town is up for re-election and you are on the fence about whether or not you will vote for him. A week before the election, you run into a close friend in a coffee shop. You know him well enough to know that his worldview is similar to yours. The subject of the election comes up. You are swayed by what he says and how he says it, even though he doesn't offer any reasons. In fact, you are so strongly influenced by the interaction that you end up voting against re-electing the mayor.

Q1

To what extent would you find this influence objectionable?

Not at all Very much so

Q2

Please tell us why you answered as you did.

Type here

Next

Voting scenario, PREAUTHORIZED, RATIONAL

Imagine that the mayor in your town is up for re-election and you are on the fence about whether or not you will vote for him. A week before the election, you run into a close friend in a coffee shop. You know him well enough to know that his worldview is similar to yours. The subject of the election comes up. You are swayed by what he says and how he says it, especially as he offers up a set of reasons. In fact, you are so strongly influenced by the interaction that you end up voting against re-electing the mayor.

Q1

To what extent would you find this influence objectionable?

Not at all Very much so

Q2

Please tell us why you answered as you did.

Type here

Next

Voting scenario, NON-PREAUTHORIZED, ARATIONAL

Imagine that the mayor in your town is up for re-election and you are on the fence about whether or not you will vote for him. A week before the election, you run into an acquaintance in a coffee shop. You don't know him well enough to know whether his worldview is similar to yours. The subject of the election comes up. You are swayed by what he says and how he says it, even though he doesn't offer any reasons. In fact, you are so strongly influenced by the interaction that you end up voting against re-electing the mayor.

Q1

To what extent would you find this influence objectionable?

Not at all Very much so

Q2

Please tell us why you answered as you did.

Type here

Next

Voting scenario, NON-PREAUTHORIZED, RATIONAL

Imagine that the mayor in your town is up for re-election and you are on the fence about whether or not you will vote for him. A week before the election, you run into an acquaintance in a coffee shop. You don't know him well enough to know whether his worldview is similar to yours. The subject of the election comes up. You are swayed by what he says and how he says it, especially as he offers up a set of reasons. In fact, you are so strongly influenced by the interaction that you end up voting against re-electing the mayor.

Q1

To what extent would you find this influence objectionable?

Not at all Very much so

Q2

Please tell us why you answered as you did.

Type here

Next

Healthy eating scenario, PREAUTHORIZED, ARATIONAL

Imagine that you update the operating system on your smartphone. The update includes a suite of new apps, including one that is intended to encourage healthy eating. The app has been publicly recommended by your local Health and Wellness Center. Because you are a health nut and have regularly used the Center's services, you are confident that its mission is aligned with your interests. The app works by flashing subliminal images of healthy food onto your screen several times a day, especially around meal times. This has a strong influence on your eating habits, even though the subliminal images do not offer you any reasons for eating more healthily.

Q1

To what extent would you find this influence objectionable?

Not at all Very much so

Q2

Please tell us why you answered as you did.

Type here

Next

Healthy eating scenario, PREAUTHORIZED, RATIONAL

Imagine that you update the operating system on your smartphone. The update includes a suite of new apps, including one that is intended to encourage healthy eating. The app has been publicly recommended by your local Health and Wellness Center. Because you are a health nut and have regularly used the Center's services, you are confident that its mission is aligned with your interests. The app works by sending you brief text messages about the benefits of healthy eating several times a day, especially around meal times. This has a strong influence on your eating habits, especially as the text messages offer you reasons for eating more healthily.

Q1

To what extent would you find this influence objectionable?

Not at all Very much so

Q2

Please tell us why you answered as you did.

Type here

Next

Healthy eating scenario, NON-PREAUTHORIZED, ARATIONAL

Imagine that you update the operating system on your smartphone. The update includes a suite of new apps, including one that is intended to encourage healthy eating. The app has been publicly recommended by your local Health and Wellness Center. Because you are not a health nut and have never used the Center's services, you don't know whether its mission is aligned with your interests. The app works by flashing subliminal images of healthy food onto your screen several times a day, especially around meal times. This has a strong influence on your eating habits, even though the subliminal images do not offer you any reasons for eating more healthily.

Q1

To what extent would you find this influence objectionable?

Not at all Very much so

Q2

Please tell us why you answered as you did.

Type here

Next

Healthy eating scenario, NON-PREAUTHORIZED, RATIONAL

Imagine that you update the operating system on your smartphone. The update includes a suite of new apps, including one that is intended to encourage healthy eating. The app has been publicly recommended by your local Health and Wellness Center. Because you are not a health nut and have never used the Center's services, you don't know whether its mission is aligned with your interests. The app works by sending you brief text messages about the benefits of healthy eating several times a day, especially around meal times. This has a strong influence on your eating habits, especially as the text messages offer you reasons for eating more healthily.

Q1

To what extent would you find this influence objectionable?

Not at all Very much so

Q2

Please tell us why you answered as you did.

Type here

Next

Voting scenario, FAMILIAR, UNKNOWN WORLDVIEW

Imagine that the mayor in your town is up for re-election and you are on the fence about whether or not you will vote for him. A week before the election, you run into a co-worker in a coffee shop. Although you have known him for many years, you haven't discussed politics and therefore don't know whether his political worldview is similar to yours. The subject of the election comes up, and you are swayed by what he says and how he says it. In fact, you are so strongly influenced by the interaction that you end up voting against re-electing the mayor.

Q1

To what extent would you find this influence objectionable?

Not at all Very much so

Q2

Please tell us why you answered as you did.

Type here

Next

Voting scenario, FAMILIAR, SHARED WORLDVIEW

Imagine that the mayor in your town is up for re-election and you are on the fence about whether or not you will vote for him. A week before the election, you run into a co-worker in a coffee shop. Because you have known him for many years, you have discussed politics and therefore know that his political worldview is similar to yours. The subject of the election comes up, and you are swayed by what he says and how he says it. In fact, you are so strongly influenced by the interaction that you end up voting against re-electing the mayor.

Q1

To what extent would you find this influence objectionable?

Not at all Very much so

Q2

Please tell us why you answered as you did.

Type here

Next

Voting scenario, UNFAMILIAR, UNKNOWN WORLDVIEW

Imagine that the mayor in your town is up for re-election and you are on the fence about whether or not you will vote for him. A week before the election, you run into a co-worker in a coffee shop. Because you haven't known him for very long, you haven't discussed politics and therefore don't know whether his political worldview is similar to yours. The subject of the election comes up, and you are swayed by what he says and how he says it. In fact, you are so strongly influenced by the interaction that you end up voting against re-electing the mayor.

Q1

To what extent would you find this influence objectionable?

Not at all Very much so

Q2

Please tell us why you answered as you did.

Type here

Next

Voting scenario, UNFAMILIAR, SHARED WORLDVIEW

Imagine that the mayor in your town is up for re-election and you are on the fence about whether or not you will vote for him. A week before the election, you run into a co-worker in a coffee shop. Although you haven't known him for very long, you have discussed politics and therefore know that his political worldview is similar to yours. The subject of the election comes up, and you are swayed by what he says and how he says it. In fact, you are so strongly influenced by the interaction that you end up voting against re-electing the mayor.

Q1

To what extent would you find this influence objectionable?

Not at all Very much so

Q2

Please tell us why you answered as you did.

Type here

Next

Common questions (Q5-Q7 are specific to the Voting scenario; comparable questions were asked of respondents in the Healthy eating scenario)

Q3

To what extent would you find this influence manipulative?

Not at all — Very much so

Q4

To what extent would you find this influence welcome?

Not at all — Very much so

Next

With respect to the situation that you were asked you to imagine on the previous page, please indicate the extent to which you agree with the following statements:

Q5

You are confident that he shares your worldview.

Strongly disagree — Strongly agree

Q6

He offered reasons for why you should vote against re-electing the mayor.

Strongly disagree — Strongly agree

Q7

To what extent is voting in a mayoral election important to you?

Not very important — Very important

Next

Comprehension Check

The scenario described on the previous pages was about which of the following (choose one):

- Earthquake
- Sporting event
- Election
- Vacation
- Healthy eating

Next

Demographics

What is your age (in years)?

Type here

Please indicate your biological sex.

- Female
- Male

What is the highest level of education you have attained?

- Some high school
- High school diploma
- Some college or university
- College or university degree
- Some post-graduate
- Post-graduate degree

What is your total annual household income?

- <$22,500
- $22,500–39,999
- $40,000–59,999
- $60,000–89,999
- $90,000 or more
- Prefer not to say

Next

Individualism – Communitarianism Scale

People in our society often disagree about how far to let individuals go in making decisions for themselves. How strongly do you agree or disagree with each of these statements?

	Strongly disagree	Moderately disagree	Slightly disagree	Slightly agree	Moderately agree	Strongly agree
The government interferes far too much in our everyday lives.	○	○	○	○	○	○
Sometimes government needs to make laws that keep people from hurting themselves.	○	○	○	○	○	○
It's not the government's business to try to protect people from themselves.	○	○	○	○	○	○
The government should stop telling people how to live their lives.	○	○	○	○	○	○
The government should do more to advance society's goals, even if that means limiting the freedom and choices of individuals.	○	○	○	○	○	○
Government should put limits on the choices individuals can make so they don't get in the way of what's good for society.	○	○	○	○	○	○

Next

Conclusion

This concludes our survey. Selecting the "submit" button will result in the submission of your survey, and provide you with a *randomly assigned* confirmation code for use in getting your reward from Amazon's Mechanical Turk. We sincerely appreciate your participation.

Submit
